# Graphene Oxide-Based Nanocomposites for Stereolithography (SLA) 3D Printing: Comprehensive Mechanical Characterization under Combined Loading Modes

**DOI:** 10.3390/polym16091261

**Published:** 2024-05-01

**Authors:** Guilherme Elias Saltarelli Garcia, Rogerio Ramos de Sousa Junior, Julia Rocha Gouveia, Demetrio Jackson dos Santos

**Affiliations:** Center for Engineering, Modeling and Applied Social Sciences, Federal University of ABC, Santo André 09210-580, Brazil; guilherme.elias@aluno.ufabc.edu.br (G.E.S.G.); juliargouveia@gmail.com (J.R.G.)

**Keywords:** additive manufacturing, graphene oxide, nanocomposite, mechanical properties, combined loadings, Drucker–Prager model

## Abstract

Additive manufacturing, particularly Stereolithography (SLA), has gained widespread attention thanks to its ability to produce intricate parts with high precision and customization capacity. Nevertheless, the inherent low mechanical properties of SLA-printed parts limit their use in high-value applications. One approach to enhance these properties involves the incorporation of nanomaterials, with graphene oxide (GO) being a widely studied option. However, the characterization of SLA-printed GO nanocomposites under various stress loadings remains underexplored in the literature, despite being essential for evaluating their mechanical performance in applications. This study aimed to address this gap by synthesizing GO and incorporating it into a commercial SLA resin at different concentrations (0.2, 0.5, and 1 wt.%). Printed specimens were subjected to pure tension, combined stresses, and pure shear stress modes for comprehensive mechanical characterization. Additionally, failure criteria were provided using the Drucker-–Prager model.

## 1. Introduction

The use of additive manufacturing (AM) techniques in the so-called Industry 4.0 has been playing a crucial role in transforming the manufacturing processes of components with complex geometries [1,2]. Often known as 3D printing, AM offers a rapid, efficient, and highly customizable production of parts. Through 3D printing methods, computer-aided design (CAD) models are transformed into diverse components made from various classes of materials, such as ceramics, metals, and polymers [3,4,5]. Most 3D printing processes for metals and ceramics involve the localized sintering of particles through the use of collimated laser or electron beams, although other techniques such as binder jetting may also be employed [6,7,8]. For polymers, the two main 3D printing techniques employed are Filament Deposition Modeling (FDM) (also known as Filament Fused Fabrication—FFF) and Stereolithography (SLA) [5,9,10].

The SLA method involves the deposition of layers of a three-dimensional solid made of photopolymerizable monomers onto different kinds of substrates with the use of ultraviolet–visible radiation [11,12,13]. Commercial SLA processes are predominantly based on photopolymerizable resins derived from acrylate, epoxy, or urethane monomers [5,14,15]. In most commercial SLA processes (also known as LCD-SLA or LCD 3D printing), a movable substrate (Z-axis) is immersed in a photopolymerizable resin container [5,11], wherein, below the resin container is positioned a high-resolution monochromatic screen, which serves as a mask, controlling the deposition of layers in the solid [11,16,17]. The screen usually permits the passage of 405 nm light (usually emitted from LED backlighting) for resin curing in the designated region of the solid’s cross-section, while effectively blocking radiation in adjacent areas. Successive layers of solid sections are deposited upside-down on the immersed substrate moving along the Z-axis [5,11]. Once the printing errors in SLA are only related to the displacement error of Z-axis motor and the resolution of the LCD screen, SLA-printed parts exhibit superior finish and dimensional accuracy compared to FDM-printed parts, which might present errors associated with motors in all three axes, as well as the printing resolution limitation and clogging issues related to the nozzle [18,19,20].

Despite its advantages in comparison to FDM 3D printing, the low mechanical properties of the typically employed photosensitive resins often limit the use of SLA to prototypes, molds, or aesthetic models. To enhance the mechanical properties and other physicochemical characteristics, and, thus, increase the added value of SLA-printed parts, additives, typically nanomaterials, are employed [15,21,22]. Among these reinforcements, graphene [5,22,23,24] and graphene oxide (GO) [14,21,25,26] stand out, although the latter is preferred in nanocomposite applications due to its better dispersibility into polymer matrices [24,27,28].

Graphene is a nanomaterial based on a single layer of carbon atoms arranged in a hexagonal honeycomb lattice. It is an allotrope of carbon that has been widely studied for various applications due to its exceptional mechanical properties and high thermal and electrical conductivity [14,24,28]. On the other hand, GO is also a two-dimensional nanomaterial based on a carbon skeleton. However, because of its oxygenated functional groups, GO presents better interaction with organic solvents, improved dispersibility in polymers, easier processability, and the ability to be easily functionalized with other chemical groups compared to graphene [29,30,31]. 

The more prominent applications of GO are in electronics, photonics, optoelectronics, sensors, drug delivery systems, and, primarily, nanocomposites [29,30,31,32,33]. In fact, various studies in the literature demonstrate an increase in the modulus of elasticity and tensile strength with the incorporation of GO at low concentrations (below 1 wt.%). Lin et al. incorporated GO into a commercial SLA resin (based on acrylate and methacrylate monomers) at concentrations of 0.2 and 0.5 wt.%. The authors produced complex geometry specimens using SLA 3D printing, which were evaluated through compression and tensile testing. The test specimens printed with the resin containing 0.2 wt.% of GO exhibited an increase of 24.9% in tensile strength, while those with 0.5 wt.% of GO showed a higher increase of 45.3% [21]. Manapat et al. incorporated GO at concentrations of 0.1, 0.5, and 1 wt.% into a commercial SLA resin and evaluated the tensile strength of the printed nanocomposites. Prior to mechanical testing, the authors conducted an annealing process at 50 and 100 °C for 12 h. They reported a 673.6% increase in the ultimate tensile strength for samples containing 1 wt.% of GO. The authors attribute this result to annealing, as it induces cross-linking via acid-catalyzed esterification and the removal of intercalated water, thereby improving filler–matrix interaction [3]. The literature indicates that the incorporation of GO into SLA resins results in printed nanocomposites with enhanced mechanical properties [14,19,22]. Most of these studies evaluate the mechanical properties of printed samples under a single stress mode, typically through tensile or compressive testing.

To the best of our knowledge, this is the first work in the literature that incorporates GO as a nanofiller into SLA resins based on acrylic monomers and assesses specimens of the same geometry under several modes of mechanical loading: pure tension, pure shear, and combined stresses. It is worth noting that employing a multi-stress testing approach represents real mechanical loading conditions, thereby offering significant advantages in elucidating the mechanical properties of a material. For this purpose, a reduced Arcan device was employed in conjunction with a universal testing machine. The Arcan device enables testing of bi-trapezoidal (‘butterfly shaped’) specimens under combined loading modes, with 0°, 45°, and 90° angles corresponding to pure tension, mixed stresses, and pure shear, respectively [34,35,36]. The comprehensive elucidation of the mechanical performance of GO-based nanocomposites is crucial to enable the application of these materials in more fields, such as structural materials for the aerospace sector, custom-shaped electrodes, and medical and dental applications [30,31]. 

## 2. Materials and Methods

### 2.1. Materials

The graphite (Grafine 996100) was acquired from the company Nacional de Grafite (Salto da Divisa, Brazil). H_2_SO_4_, H_3_PO_4_, HCl, and acetone were purchased from Labsynth (Diadema, Brazil), while KMnO_4_ and H_2_O_2_ used in the synthesis of GO were acquired from Sigma-Aldrich (São Paulo, Brazil). The GO-based nanocomposites were prepared using a standard rigid acrylic photosensitive resin obtained from Shenzhen Creality 3d Technology Co. (Shenzhen, China). This acrylic resin is transparent, with a dynamic viscosity of 150–200 mPa.s, density of 1.15 g.cm^–3^, and an absorption band between 355 and 410 nm [37]. All reagents were used as purchased.

### 2.2. Synthesis of Graphene Oxide

The synthesis of GO nanoparticles was carried out based on the procedure proposed by Marcano et al. [38]. In summary, 3 g of graphite was added to a mixture of H_2_SO_4_/H_3_PO_4_ (9:1 *v:v*) and maintained under constant agitation at room temperature. Then, 18 g of KMnO_4_ was slowly added to the mixture. The mixture was heated to 50 °C and maintained under constant agitation for 12 h. Subsequently, the reaction was cooled to room temperature, poured into 400 mL of ice, and 3 mL of 30% H_2_O_2_ was added. The reaction was centrifuged at 4000 rpm for 4 h. The solid was then successively washed in 200 mL of distilled water and 30% HCl solution, followed by two washes in ethanol. At each washing step, the mixture was centrifuged at 4000 rpm for 4 h. Finally, the solid was coagulated in 200 mL of ether and dried under vacuum at 80 °C overnight. 

### 2.3. Characterization of GO

Pristine graphite and GO were vacuum-dried overnight prior to the characterization steps. X-ray diffraction (XRD) patterns were obtained using a Bruker D8-Focus X-ray diffractometer (Billerica, MA, USA) with a CuKα (λ = 1.54 Å) radiation source, in the 2θ range of 5–30°, with a scan rate of 1°/min.

Infrared spectroscopy operating in attenuated total reflection mode (FTIR-ATR) measurements were conducted using Perkin Elmer Spectrum Two equipment (Hopkinton, MA, USA). A total of 64 scans were performed over the range of 4000 to 500 cm^−1^, with a resolution of 4 cm^−1^.

### 2.4. GO-Based SLA Resin Preparation

The procedure for dispersing GO in the acrylic resin was based on the works of Manapat et al. [3] and Palaganas et al. [19]. GO was dispersed in acetone with sonication assistance for 30 min at room temperature. Subsequently, the GO dispersion was added to the resin with magnetic stirring for 30 min. Finally, this mixture was maintained under constant agitation for 12 h at 50 °C for complete solvent evaporation. 

The specimens were produced using an Anycubic Photon SLA 3D printer (Shenzhen, China), operating at a wavelength of 405 nm to cure the layers of the printed parts. The parts were printed with an exposure time of 120 s for the first six layers (bottom layers) and 45 s for subsequent layers. A 100% infill density was utilized. The printed specimens were placed in a post-printing chamber equipped with 405 nm LEDs for 2 h and then subjected to a vacuum oven at a temperature of 90 °C for 24 h to complete their cross-linking process.

### 2.5. Characterization of GO-Based Nanocomposites

Optical microscopy was conducted to assess the dispersion of GO in the resin prior to the curing process. A small amount of each sample was deposited between glass slides and lightly pressed using tweezers. Micrographs were taken using a Carl Zeiss Axio Scope A1 optical microscope (Oberkochen, Germany). The particle size analysis was conducted using ImageJ software version 1.54, and the results were expressed as confidence intervals (at 95%).

The viscosity of the mixtures was evaluated using a rotational rheometer Anton Paar MCR 502 (Graz, Austria), employing a parallel plate geometry of 25 mm, with a shear rate scan ranging from 1 to 100 s^−1^ at room temperature.

After the curing of the samples, the viscoelastic properties of the GO-based nanocomposites and pure acrylic resin were assessed by dynamic mechanical analysis (DMA) on a rotational rheometer Anton Paar MCR 502 (Graz, Austria). Analyses were performed on rectangular samples with dimensions of 40 × 10 × 2 mm, in torsion mode at a frequency of 1 Hz and shear strain of 0.5%. Temperature ramp was conducted from 25 to 140 °C at a heating rate of 3 °C/min.

**Figure 1:**
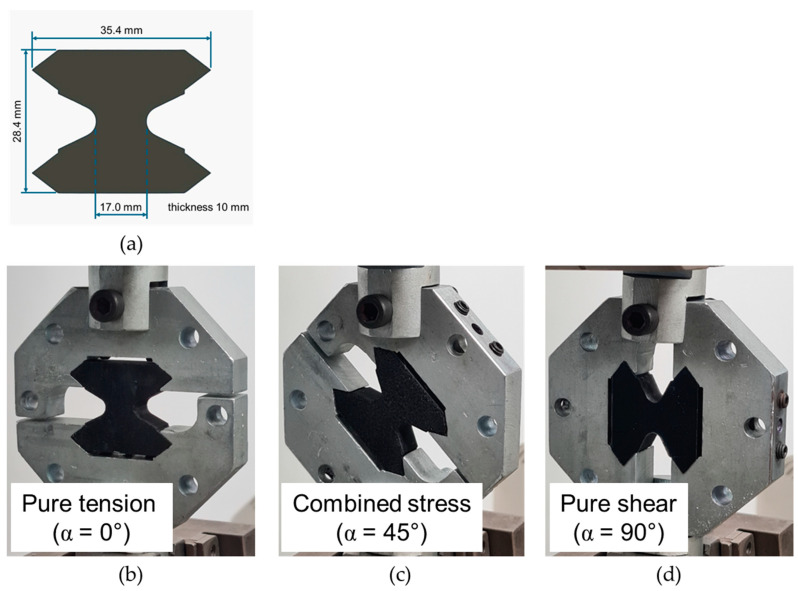
(**a**) Schematic drawing of the test specimen and (**b**–**d**) mechanical test modes.

Mechanical tests under different loading modes were performed using a modified Arcan device [35,39]. Trapezoidal samples were printed with gauge section dimensions of 17 mm width and 10 mm thickness. The schematic drawing of the test specimen is presented in Figure 1a. Arcan tests were conducted on an Instron 3369 universal mechanical testing machine (Norwood, MA, USA), with a load cell of 50 kN, and a displacement rate of 2 mm/min. Tests were performed at angles of 0° (pure tension), 45° (combined stress), and 90° (pure shear). Figure 1 shows the assembled Arcan device and the mechanical test modes.

The fracture surfaces of the pure tension tests were coated with a thin layer of gold (15 nm) and evaluated by scanning electron microscopy (SEM), using Jeol JSM-6010LA equipment (Akishima, Japan) with an acceleration energy of 15 kV.

### 2.6. Failure Criterion

The mechanical results of normal stress (*σ_n_*) and shear stress (*τ_s_*) at failure, maximum strength, were obtained from the measurements of the nominal stress component (*σ*) from the multiple load tests using the Arcan device, at their respective loading angles (*α*). Equations (1) and (2) are used to determine *σ_n_* and *τ_s_*, respectively:

(1) $$\sigma_{n}=\sigma cos\left(\alpha \right)$$

(2) $$\tau_{s}=\sigma sin\left(\alpha \right)$$

Based on the experimental data, a failure envelope was obtained to predict the failure of GO-based nanocomposites, based on the Drucker–Prager theoretical model. The Drucker–Prager model was initially developed for geomaterials based on elastoplasticity, considering deviatoric and hydrostatic stress components [34]. In recent years, the model has been satisfactorily applied in predicting the failure criterion of polymer materials, including composites, adhesives, and adhesive joints [34,36,39,40].

The Drucker–Prager failure envelope is described by Equations (3)–(7):

(3) $$\left(\frac{\lambda -1}{2\lambda }\right)\times I_{1}+\sqrt{\frac{3}{\lambda }\times J_{2}+{\left(\frac{\lambda -1}{2\lambda }\right)}^{2}\times {I_{1}}^{2}}-\sigma_{F}=0$$

(4) $$\lambda =\left(\frac{1}{6}\right)\times \left(\beta^{2}+\beta \times \sqrt{\beta^{2}+12}+6\right)$$

(5) $$\beta =3\times \left(\frac{1-\nu }{1+\nu }\right)\times \left(\frac{\tau_{s}}{\sigma_{n}}\right)-\left[\frac{{\left(1-2\nu \right)}^{2}}{\left(1-\nu \right)\times \left(1+\nu \right)}\right]\times \left(\frac{\sigma_{n}}{\tau_{s}}\right)$$

(6) $$I_{1}=\sigma_{x}+\sigma_{y}+\sigma_{z}$$

(7) $$J_{2}=\frac{\left[{\left(\sigma_{x}-\sigma_{y}\right)}^{2}+{\left(\sigma_{y}-\sigma_{z}\right)}^{2}+{\left(\sigma_{z}-\sigma_{x}\right)}^{2}+6\times \left(\tau_{xy}^{2}+\tau_{yz}^{2}+\tau_{zx}^{2}\right)\right]}{6}$$

where *I*_1_ and *J*_2_ are the first invariant of the stress tensor and the second invariant of the stress deviator, respectively. *λ* is the contribution to the hydrostatic pressure of the material and *ν* is the material’s Poisson coefficient. The value of *ν* = 0.4 for the acrylic resin was used based on the work of Santos and Batalha [36].

## 3. Results and Discussions

### 3.1. Characterization of GO

**Figure 2:**
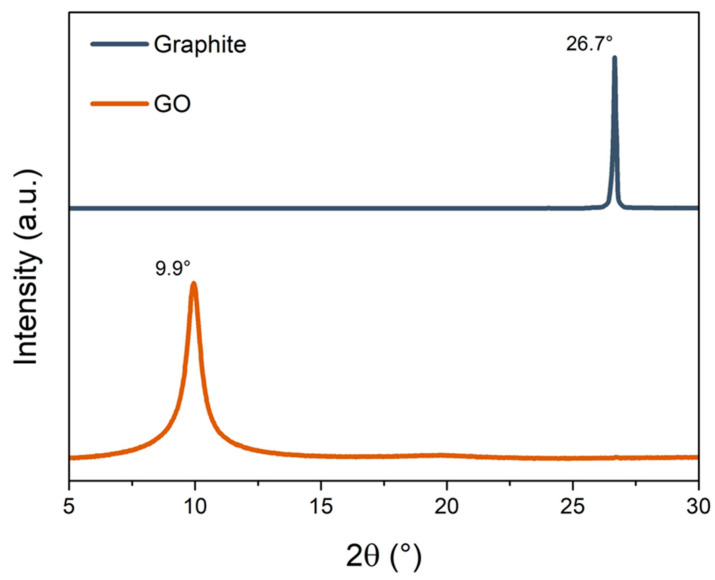
XRD pattern of pristine graphite and graphene oxide (GO).

The XRD pattern of pristine graphite and GO is presented in Figure 2. Graphite exhibits a typical peak of the (002) plane at 2θ = 26.7°, corresponding to an interplanar distance (d) of approximately 3.43 Å according to Bragg’s Law [41]. Meanwhile, the (002) plane of GO has shifted to 2θ = 9.9°, resulting in a larger interplanar distance (d ≈ 8.96 Å) when compared to graphite. This occurs due to the insertion of oxygen functional groups into the interlayer galleries of GO [42]. 

**Figure 3:**
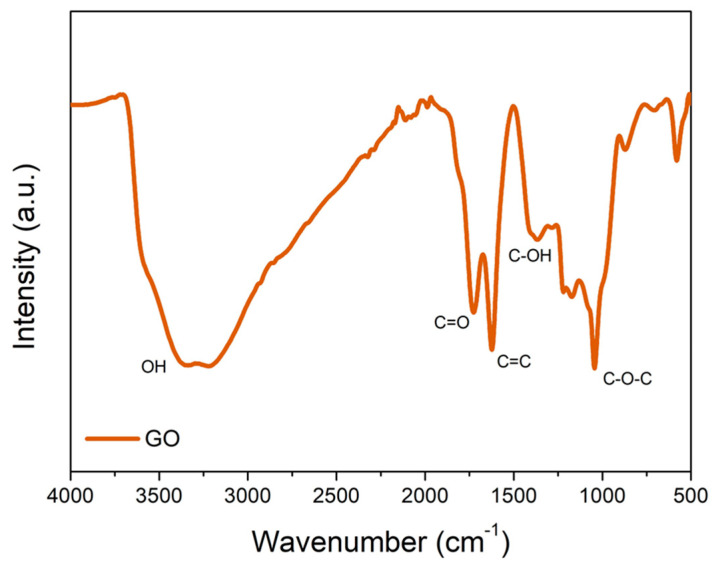
FTIR spectra of graphene oxide (GO).

The FTIR spectrum of GO (Figure 3) indicates the presence of characteristic groups of the nanomaterial. Typical intensity peaks of GO are present at 1724 cm^−1^, associated with the stretching vibration of the C=O bond, 1622 cm^−1^ from the C=C bond present in the aromatic ring, 1367 cm^−1^ from the stretching of C-OH, and the peak region at 1041 cm^−1^ associated with the epoxide C-O-C. The broad band, between 3000 and 3500 cm^−1^, is associated with hydroxyl group stretching vibrations [42,43,44]. This result, together with observations from XRD, demonstrates the successful synthesis of GO used in this work.

### 3.2. GO Dispersion

**Figure 4:**
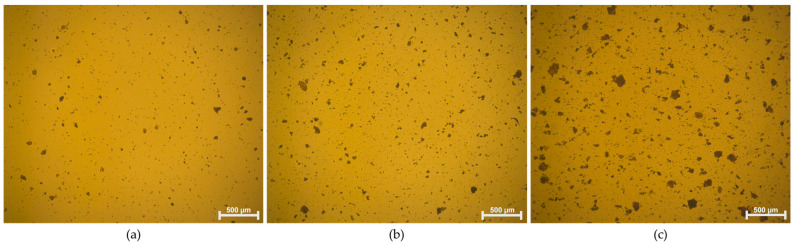
Optical micrographs of (**a**) 0.2 GO, (**b**) 0.5 GO, and (**c**) 1.0 GO.

Micrographs obtained from an optical microscope are an important technique that allows for the rapid analysis of dispersed phases in polymers [45,46]. Figure 4 presents the micrographs obtained for samples with different concentrations of GO in the resin. As expected, the acrylic resin exhibits a significant interaction with the functional groups of GO [3,14], resulting in a relatively uniform dispersion of GO particles, particularly at lower concentrations of GO. The 0.2 GO sample (Figure 4a) shows well-distributed GO with the formation of small aggregates, with an average size of 11.38 ± 1.38 μm. In contrast, sample 0.5 GO (Figure 4b) exhibits a higher number of aggregates with a larger average size of 14.3 ± 2.12 μm. At the higher concentration of GO (1.0 GO—Figure 4c), even larger aggregates are formed, reaching sizes on the order of 160 μm and an average size of 21.14 ± 3.28 μm. The formation of aggregates at higher concentrations of nanomaterials is consistent with previous findings in the literature [3,14,22], suggesting that higher concentrations of GO promote stronger interactions between the nanomaterials through van der Waals forces and π-π bonding [47].

**Figure 5:**
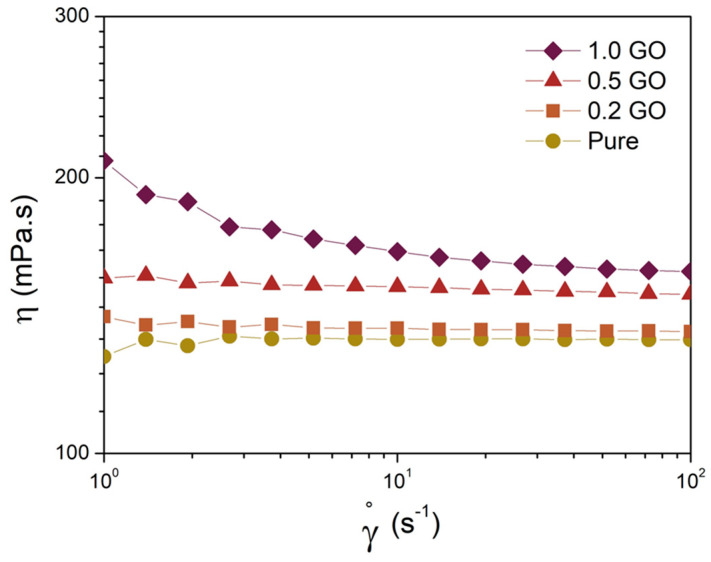
Viscosity as a function of shear rate of neat resin and GO-based samples.

Viscosity is a crucial factor for the efficiency of UV curing, with an optimal viscosity range typically falling between 200 and 1000 mPa·s [17]. If the viscosity is too high, it takes longer for the resin to flow and fill the volume corresponding to the cured layer. This extended time can exceed the exposure time of the next layer, leading to voids and defects that compromise the printed parts’ finish and their mechanical properties. Figure 5 illustrates the viscosity curves of the resin at different concentrations of GO, demonstrating a rise in viscosity as the concentration of GO increases, reflecting the strong interaction between the resin and the functional groups of GO [14]. Nevertheless, the viscosity values are within the ideal range for application. Specifically, at a shear rate of 1 s^−1^, the viscosity of the pure resin measures 127 mPa·s, while that of the sample containing 1.0 wt.% GO is 208 mPa·s. Furthermore, the pure resin, as well as the samples containing 0.2 and 0.5 wt.% of GO, exhibit Newtonian behavior across the entire range of shear rates. In contrast, the sample containing 1 wt.% of GO displays non-Newtonian rheological behavior at low shear rates, likely due to the presence of larger GO aggregates [47], as observed in the optical microscopy images (Figure 4).

### 3.3. GO-Based Nanocomposites

#### 3.3.1. Viscoelastic Properties

**Figure 6:**
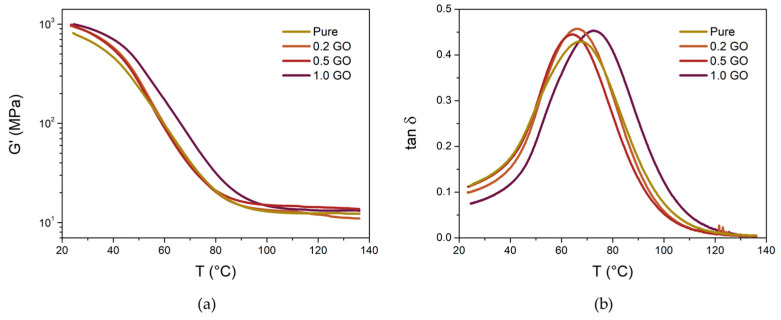
(**a**) G’ and (**b**) tan δ curves of GO-based nanocomposites.

After the UV curing process, the samples were subjected to DMA analysis (Figure 6). Figure 6a demonstrates the storage modulus (G’) curves, while Figure 6b shows the damping factor (tan δ = G”/G’) curves. The G’ curve presents consistent behavior across all sample conditions, with an initial high value in lower temperatures followed by a significant decrease attributed to the structural mobility of the polymeric matrix, resulting in a G’ plateau at approximately 100 °C. This plateau, known as the rubbery plateau (G_N_), directly correlates with the sample’s crosslink density [48]. Notably, there is no proportional variation in the plateau modulus among the samples. The G’ values at 120 °C are detailed in Table 1. On the one hand, the 0.2 GO sample demonstrates a G′ plateau slightly lower than that of the pure resin (12.0 MPa compared to 12.6 MPa). On the other hand, at higher GO concentrations, the 0.5 GO sample exhibits the highest G_N_ value, 14.3 MPa. At the end, the 1.0 GO sample shows a slight decrease in the G_N_, 13.2 MPa, compared to the 0.5 wt.% GO concentration. Nonetheless, it maintains a higher value relative to the pure resin. This behavior may be attributed to two concomitant phenomena: (i) the addition of nanomaterials reduces the depth of cure of the samples [14,20], consequently impacting crosslink density; (ii) the incorporation of the reinforcement phase intrinsically enhances the stiffness of the system, resulting in higher G’ values [45,49,50]. Therefore, the G’ value at the rubbery plateau might be a consequence of the balance between these two phenomena.

The tan δ curves indicate the structural mobility of the material, often correlating with its energy dissipation [51], with their peak typically corresponding to the glass transition temperature (T_g_) of the sample. The T_g_ values are provided in Table 1. It is evident that at low concentrations of GO, there is minimal variation in T_g_ compared to the neat resin, with a slight tendency to decrease. This observation supports the hypothesis of reduced crosslink density in the samples due to the addition of GO, as evidenced by the G’ values at low GO concentrations. In contrast, the sample containing 1.0 wt.% GO exhibits a T_g_ value 5.3 °C higher than the neat resin. This increase in T_g_ at higher GO concentrations suggests that the presence of nanomaterials hinders the mobility of the polymer chain, resulting in an elevated T_g_ [52]. Finally, all nanocomposites demonstrate slightly higher tan δ intensity compared to the neat resin, attributed to the increased energy dissipation rate at the polymer–GO interface [52,53].

#### 3.3.2. Combined Load Experiments and Failure Criteria

Combined load experiments, such as those conducted using the Arcan device, enable the acquisition of multidirectional data, facilitating a broader and more precise characterization of the mechanical behavior of polymeric materials. Table 2 presents the nominal rupture stress data (*σ*) obtained at multidirectional angles of 0°, 45°, and 90°, as well as the contributions of normal stress (*σ_n_*) and shear stress (*τ_s_*) derived from Equations (1) and (2), respectively.

Comparing the mean values of σ within the same sample condition (content of GO), at different loading angles, it becomes apparent that the values fall within the same range, consistent with findings reported in a hybrid composite system [40]. Additionally, when analyzing samples with varying concentrations of GO, two distinct trends emerge: (i) samples with low GO concentration (0.2 wt.%) exhibit lower mechanical strength than the neat resin; (ii) higher concentrations of GO (0.5 and 1.0 wt.%) demonstrate superior mechanical strengths. In the former case, as previously noted, the addition of GO reduces the curing depth of the sample, and the low GO concentration was insufficient to mitigate this effect. In the latter case, at higher concentrations of GO, the mechanical reinforcement effect of the nanomaterial predominates, especially with a more even dispersion. This is evidenced by the 0.5 GO samples having higher mechanical strengths compared to the 1.0 GO samples.

Although experimental data under combined loadings allow for an initial analysis of the mechanical behavior of nanocomposites, applying these data to a theoretical model enables the determination of rupture strength values in all loading directions. For this purpose, we employed the Drucker–Prager model, which delineates a failure envelope across all stress and shear loading combinations.

**Figure 7:**
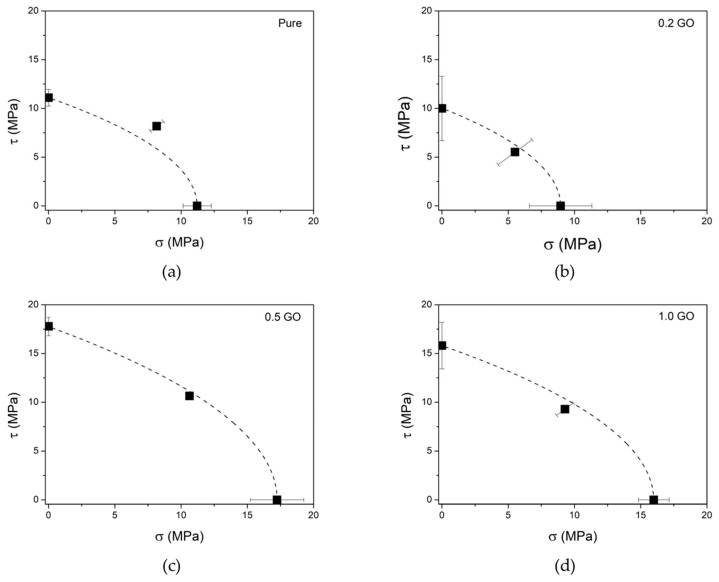
Failure envelope according to Drucker–Prager models (dashed lines) and average experimental results (black dots) of (**a**) pure resin, (**b**) 0.2 GO, (**c**) 0.5 GO, and (**d**) 1.0 GO.

Figure 7 presents the mean experimental data at different concentrations of GO, accompanied by the failure envelope (represented by a dashed line) obtained from the Drucker–Prager model. It is evident that the pure resin does not exhibit good convergence with the Drucker–Prager theoretical model. However, the model’s convergence is satisfactory for GO-based nanocomposites. One characteristic of the Drucker–Prager model is its consideration of the hydrostatic pressure component of the theoretical model [54], making it particularly suitable for describing polymer materials with homogeneous plastic deformation [55], while pure resin tends to exhibit brittle behavior.

#### 3.3.3. Fracture Surface

**Figure 8:**
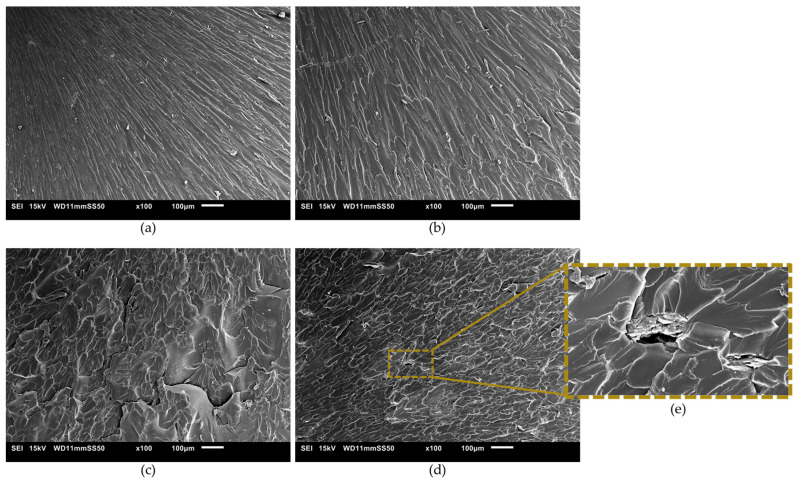
SEM of fracture surface of (**a**) pure resin, (**b**) 0.2 GO, (**c**) 0.5 GO, (**d**) 1.0 GO, and (**e**) magnified view of the 1.0 GO sample.

Figure 8 presents the SEM micrographs obtained for the GO-based nanocomposites. Consistent with observations from optical microscopy, low concentrations of GO do not exhibit aggregate formation, while such aggregates are evident in samples containing 1.0 wt.% of GO (Figure 8d,e). It is also noteworthy to observe the change in the deformation pattern on the sample surface as a function of GO concentration. With the addition of the nanomaterial, a rougher pattern formed on the fracture surface of the samples, accompanied by the development of plastic flow bands, especially at higher concentrations of GO. This phenomenon occurs because cracks are affected by the presence of the nanomaterial during their propagation. GO acts as a barrier to crack growth and promotes crack branching, ultimately leading to the formation of new fracture surfaces and enhancing the overall toughness of the sample [14,56]. The modification in the fracture surface morphology of the nanocomposites may be attributed to the improved convergence observed between experimental data and the Drucker–Prager theoretical model.

## 4. Conclusions

This study comprehensively evaluated the mechanical properties of SLA-printed nanocomposites under combined loading tests, utilizing a commercial SLA resin incorporated with varying concentrations of graphene oxide (GO) at 0.2, 0.5, and 1.0 wt.%. Analysis of GO dispersion in the resin revealed the tendency to form larger aggregates at concentrations of 1.0 wt.% GO. Nevertheless, all samples exhibited viscosity ranges suitable for additive manufacturing. Mechanical testing, conducted with an Arcan device, revealed that samples containing a low GO concentration (0.2 wt.%) exhibited inferior mechanical strength compared to the pure resin. Conversely, higher GO concentrations (0.5 wt.% and 1.0 wt.%) demonstrated superior mechanical strengths. Additionally, viscoelastic analysis of the nanocomposites indicated an increasing trend in storage modulus (G’) with higher GO concentrations. The highest G’ value was observed at 0.5 wt.% GO, demonstrating improved dispersion within the polymer matrix and suggesting an optimization between UV matrix curing and GO stiffness.

Combined loading tests were conducted using an Arcan device, employing pure tension, combined stress, and pure shear modes. The failure envelope, enabling failure analysis in all loading directions, was obtained from experimental data using the Drucker–Prager theoretical model. While the pure resin sample showed a small divergence with the theoretical model, nanocomposites demonstrated a good fit. This observation was attributed to the improved toughness of the samples with added GO, as also evidenced by the analysis of the fracture surface morphology of the nanocomposites, allowing better convergence with the Drucker–Prager model due to the hydrostatic pressure component of the theoretical model.

Therefore, this study demonstrates the effectiveness of utilizing the Drucker–Prager theoretical model to determine the failure envelope of SLA-printed GO-based nanocomposites. These results not only contribute to the advancement of additive manufacturing of nanocomposites but also present opportunities for their application in new fields, drawing on insights gained from their mechanical behavior under different loading conditions.

## Figures and Tables

**Table 1:** Values of storage modulus (G’) and T_g_ from DMA.

Sample	G’ at 120 °C (MPa)	T_g_ (°C)
Pure	12.6	67.5
0.2 GO	12.0	66.2
0.5 GO	14.3	64.2
1.0 GO	13.2	72.8

**Table 2:** Nominal (*σ*), normal (*σ_n_*), and shear stress (*τ_s_*) of GO-based nanocomposites in combined load from Arcan device.

Sample	Angle (°)	*σ* (MPa)	*σ_n_* (MPa)	*τ_s_* (MPa)
Pure	0	11.22 ± 1.06	11.22 ± 1.06	0
	45	11.55 ± 0.71	8.17 ± 0.50	8.17 ± 0.50
	90	11.10 ± 0.85	0	11.10 ± 0.85
0.2 GO	0	8.96 ± 2.36	8.96 ± 2.36	0
	45	7.80 ± 1.82	5.52 ± 1.29	5.52 ± 1.29
	90	10.00 ± 3.29	0	10.00 ± 3.29
0.5 GO	0	17.25 ± 2.02	17.25 ± 2.02	0
	45	15.05 ± 0.32	10.64 ± 0.22	10.64 ± 0.22
	90	17.78 ± 0.94	0	17.78 ± 0.94
1.0 GO	0	15.99 ± 1.16	15.99 ± 1.16	0
	45	13.13 ± 0.89	9.28 ± 0.63	9.28 ± 0.63
	90	15.81 ± 2.37	0	15.81 ± 2.37

## Data Availability

The data presented in this study are available in this article.
